# Combination of Stimulated Thyroglobulin and Antithyroglobulin Antibody Predicts the Efficacy and Prognosis of ^131^I Therapy in Patients With Differentiated Thyroid Cancer Following Total Thyroidectomy: A Retrospective Study

**DOI:** 10.3389/fendo.2022.857057

**Published:** 2022-04-06

**Authors:** Mengjiao Pan, Zhuyao Li, Meng Jia, Xiubo Lu

**Affiliations:** Department of Thyroid Surgery, The First Affiliated Hospital of Zhengzhou University, Zhengzhou, China

**Keywords:** differentiated thyroid carcinomas, stimulated thyroglobulin, thyroid-stimulating hormone, antithyroglobulin antibody, iodine ^131^I therapeutic, predictive value

## Abstract

**Background and Purpose:**

This study aimed to analyze the diagnostic ability of the combination of stimulated thyroglobulin (sTg) and antithyroglobulin antibody (TgAb) in predicting the efficacy and prognosis of radioactive iodine (^131^I) therapy (RAIT) in patients with differentiated thyroid carcinomas (DTCs) after total thyroidectomy (TT).

**Methods:**

This retrospective study comprised 409 DTC patients who underwent ^131^I treatment following TT in the First Affiliated Hospital of Zhengzhou University from January 2019 to August 2020, and they were followed up to November 2021. Patients were divided into the successful ablation and the unsuccessful ablation group based on the classification of the efficacy of RAIT in the 2015 American Thyroid Association guidelines. The clinical characteristics and the efficacy of the initial RAIT were evaluated. The cutoffs of preablation sTg, sTg/thyroid-stimulating hormone (TSH) ratio, and sTg×TgAb product were calculated to predict the efficacy of RAIT. Univariate and multivariate logistic regression analyses were used to identify the independent risk factors for unsuccessful ablation. Kaplan–Meier curves were used to estimate the prognostic value of sTg×TgAb product affecting progression-free survival (PFS).

**Results:**

The cohort consisted of 222 cases in the successful ablation group and 187 cases in the unsuccessful ablation group. Between the two groups, preablation sTg, sTg/TSH ratio, and sTg×TgAb product were significantly higher in the unsuccessful ablation group. The area under the curve (AUC) of the sTg×TgAb product was the highest among the above three factors. The cutoffs for the worse therapeutic effect of the initial RAIT in sTg, sTg/TSH ratio, and sTg×TgAb were >2.99 ng/ml, >0.029 mg/IU, and >34.18, respectively. STg >2.99 ng/ml and sTg×TgAb product >34.18 were independent risk factors for unsuccessful ablation. Patients with sTg×TgAb product >34.18 had shorter PFS than that of patients with sTg×TgAb product ≤34.18. In separate analyses of TgAb-negative and TgAb-positive subgroups, higher sTg×TgAb was both associated with a lower success rate of RAIT and a shorter PFS.

**Conclusion:**

STg×TgAb product predicted the efficacy and prognosis of ^131^I therapy for both TgAb-negative and TgAb-positive DTC patients before the initial ^131^I treatment following TT. Thus, it can be used as a clinical reference indicator for the surveillance of DTC patients.

## Introduction

Differentiated thyroid carcinoma (DTC) is the most common thyroid malignant tumor. Presently, the standard treatments for DTC include surgery, thyroid-stimulating hormone (TSH) suppression therapy, and selective radioactive iodine-131 treatment (1, 2). Also, selective postoperative radioiodine (^131^I) treatment might improve prognosis (3). Thyroglobulin (Tg) is a widely used biomarker in the follow-up of DTC patients after thyroidectomy that assists in the diagnosis of persistent disease, distant metastasis, or disease recurrence (4, 5). The stimulated thyroglobulin (sTg) is the Tg value measured after thyroidectomy without or stopped treatment with thyroid hormone (TSH >30 mU/L) (6). Several studies indicated that the sTg before ^131^I treatment has predictive value for the efficacy of the treatment (7–12). However, sTg can be affected by TSH levels, antithyroglobulin antibody (TgAb) levels, residual thyroid, metastatic lesions, and other factors, and the impact of these factors cannot be excluded.

Antithyroglobulin antibody is an autoantibody against Tg, and the detection rate of DTC patients exceeds 25% (13). TgAb can limit the utility of Tg in the surveillance of DTC patients by interfering with the accuracy of the determination of Tg (14–16) and accelerating its clearance when Tg is complexed with TgAb (17), no matter if the status of TgAb is negative or positive (18, 19). However, the effects of TgAb on sTg have not yet been solved.

This study aimed to use TgAb to correct the predictive value of the sTg to see whether sTg×TgAb product can be a predictive index of the efficacy of radioactive iodine therapy (RAIT) and whether it can predict the prognosis of ^131^I therapy, irrespective of the positive or negative TgAb status.

## Patients and Methods

### Patients

In this retrospective study, we reviewed the medical record of DTC patients who underwent total thyroidectomy (TT) and received ^131^I treatment 4 weeks after surgery between January 2019 and August 2020 at the First Affiliated Hospital of Zhengzhou University, Zhengzhou, China. The inclusion criteria were as follows: (a) cases with histological confirmation of DTC; (b) patients with 4 weeks of levothyroxine (L-T4) withdrawal with a low iodine diet after TT; (c) patients with sTg, TgAb, and TSH outcomes before postoperative ^131^I treatment; (d) patients who stopped L-T4 for 4 weeks and underwent sTg and TgAb measurement, cervical ultrasound (US), chest computed tomography (CT) scan, whole-body scan (WBS), and single-photon emission computed tomography-computed tomography (SPECT-CT) 6 months after initial RAIT; and (e) patients with positive TgAb status (TgAb >115 kU/L) should have a substantial value. The exclusion criteria were as follows: (a) patients with partial thyroidectomy; (b) patients lost to follow-up; (c) iodine refractory thyroid cancer patients; and (d) cases with undetectable sTg or TSH or TgAb (below or above the detection limit).

### Data Collection

The following data were collected: gender; age; histopathological type; tumor size; unilateral or bilateral primary foci; tumor stage; with or without extrathyroidal extension; with or without lymph node (LN) metastasis; risk stratification of recurrence; the initial RAIT dose; sTg, TSH, and TgAb measurements before the initial ^131^I ablation; results of reexamination 6 months after initial ablation, including clinical and laboratory assessments; and progression-free survival (PFS) and follow-up duration in months. TSH, sTg, and TgAb were measured using an electrochemiluminescence immunoassay (Cobas, Germany), and all of the patients used the same method.

### ^131^I Treatment and Follow-Up

Patients were forbidden to take LT4 and strictly followed a low-iodine diet after TT for 4 weeks to achieve the goal TSH of >30 mU/L before RAIT. TSH, sTg, and TgAb measurements, cervical US, and chest CT scan were completed 3 days before RAIT. The treatment dose of RAIT was referred to as the specific disease extent of every patient according to the recommendations of the 2015 American Thyroid Association (ATA) guidelines and Chinese Thyroid Association guidelines on the management of DTC (6, 20). WBS and SPECT-CT were performed 3–5 days after ^131^I treatment. Patients started to receive TSH suppression therapy when the ^131^I treatment was completed. After 6 months of the initial ^131^I treatment, patients were required to stop taking LT4 for 4 weeks and undergo the reexamination similar to the initial RAIT. DTC patients who received the initial RAIT after the TT and the reexamination in our hospital 6 months after the initial RAIT were all followed up. After the reexamination, patients were followed up through regular check-ups, including serum-free triiodothyronine (FT3), free thyroxine (FT4), TSH, Tg, and TgAb measurements every 3 months, and the cervical US every 6 months, until November 2021. The end event was the recurrence or metastasis of DTC.

### Grouping Method

According to the 2015 ATA guidelines (6), the clinical therapeutic results of patients who underwent the initial ^131^I treatment after TT were divided into four categories: excellent response (ER), indeterminate response (IDR), biochemical incomplete response (BIR), and structural incomplete response (SIR). Based on the results of the serological and imaging examinations 6 months after the initial RAIT and the above categories, patients with ER were included in the successful ablation group, while patients with IDR, BIR, or SIR were included in the unsuccessful ablation group. To be more specific, patients were considered to have a successful ablation when subjects met the following four conditions: (a) serum suppressed Tg <0.2 ng/ml or sTg <1 ng/ml; (b) absence of radioactive iodine uptake in thyroid bed on radioactive iodine scanning; (c) TgAb levels stable or declining; and (d) absence of structural or functional evidence of disease. After evaluating the efficacy and prognosis of RAIT, we divided the patients into TgAb-positive group (TgAb >115 kU/L) and TgAb-negative group (TgAb ≤115 kU/L) according to the TgAb levels measured before the initial RAIT to further analyze the predictive value of the sTg×TgAb product in patients with different TgAb status, respectively.

### Statistical Analyses

Data were analyzed using the IBM SPSS 25.0 statistical software (IBM Corp, Armonk, NY, USA). Categorical variables were expressed as numbers with percentages, and the differences between groups were evaluated by the Chi-square test. The quantitative data in this study did not conform to a normal distribution and were represented by medians with interquartile ranges; the comparisons between groups were analyzed by the Mann–Whitney *U* test, and Z-test was used to determine the *p*-value. The Spearman’s correlation test was used to assess the correlation between sTg and TgAb. The receiver operating characteristic (ROC) curve analysis determined the best cutoff values of preablation sTg, sTg/TSH ratio, and sTg×TgAb product for predicting the unsuccessful ablation of patients and assessed the predictive values of the above variables. The ROC curves of the three predictors were compared using MedCalc version 20.009 software. The ROC curve analysis was also used to evaluate the predictive value of sTg×TgAb product in the TgAb-positive and the TgAb-negative groups, respectively. Univariate and multivariate logistic regression analyses were performed to assess the independent risk factors of successful ablation. Any variable with a *p*-value <0.1 in the univariate analysis was included in the multivariate regression analysis using forward selection procedures. Kaplan–Meier curves were used to estimate the prognostic value of different factors affecting PFS. The statistical comparisons were carried out using the log-rank test. A *p*-value <0.05 was considered statistically significant.

## Results

### Clinical Evaluation of ^131^I Treatment

**Figure 1:**
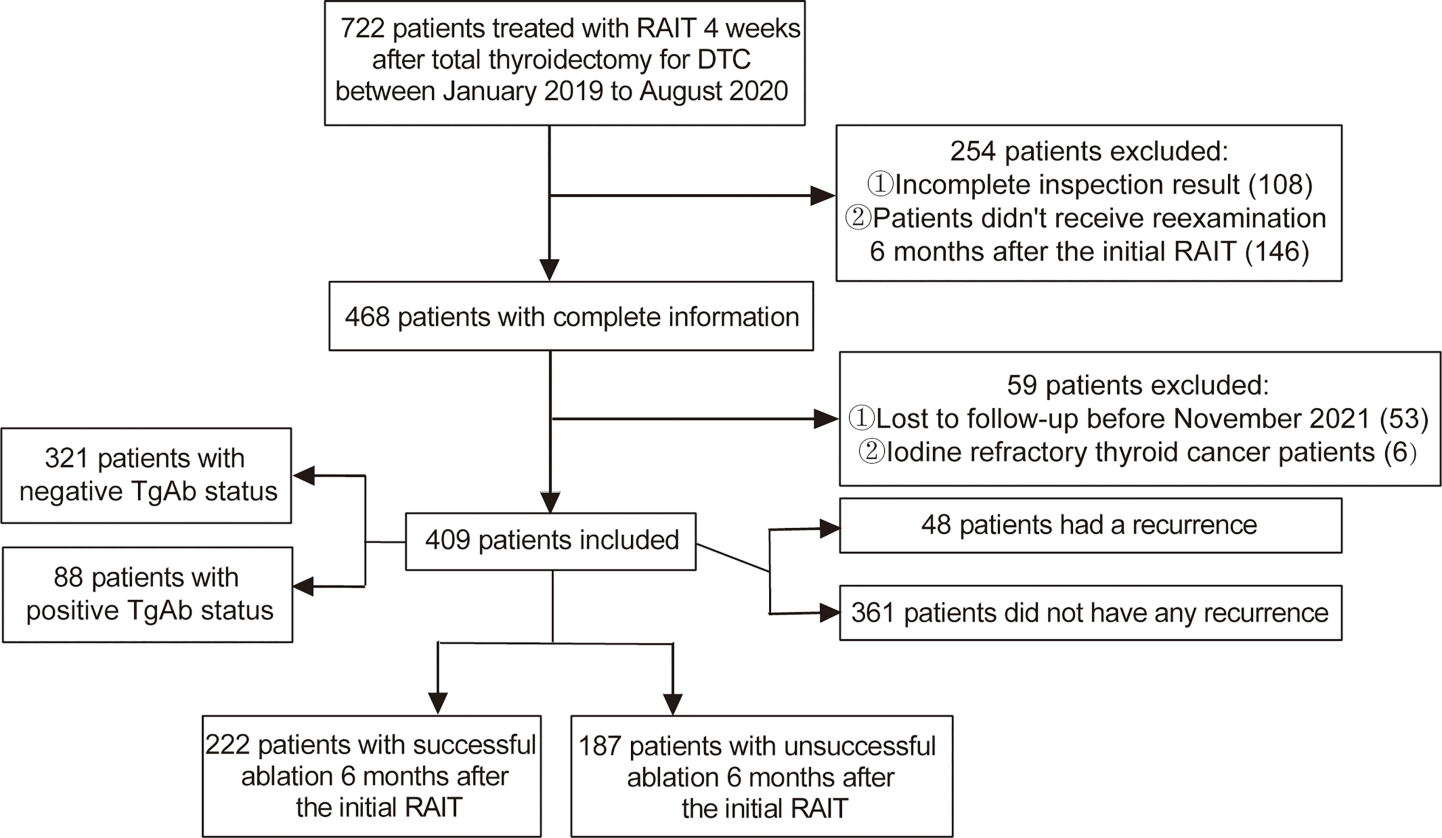
Flowchart of patient inclusion in the study.

After screening according to the inclusion and exclusion criteria, a total of 409 DTC patients were included in this retrospective study (**Figure 1**). The cohort comprised 222 (54.28%) cases that had a successful ablation, while 187 (45.72%) cases had an unsuccessful ablation. Among them, 48 (11.74%) cases had a recurrence, including 41 cases with a cervical lymph node metastasis and 7 cases with a distant metastasis. The preablation TgAb-positive group consisted of 88 (21.52%) patients, while TgAb-negative group had 321 (78.48%) patients. The median follow-up period was 23 months (IQR, 18–26 months). Correlation analysis between sTg and TgAb before the first ^131^I treatment showed that sTg and TgAb were negatively correlated (*r* = −0.474, *p* **<** 0.001). Tumor size, RAIT dose, preablation sTg, sTg/TSH ratio, and sTg×TgAb product of patients with successful ablation were significantly lower than those of patients with unsuccessful ablation (all *p* **<** 0.05). The risk stratification of recurrence of the successful ablation group was significantly different from that of the unsuccessful ablation group; intermediate- to high-risk stratification of recurrence was significantly associated with unsuccessful ablation (*p* < 0.05). Conversely, gender, age, pathological type, unilateral or bilateral primary foci, tumor stage, extrathyroidal extension, LN metastasis, TSH level, and TgAb level did not display any significant differences between the successful and unsuccessful ablation subgroups (all *p* > 0.05) (**Table 1**).

**Table 1:** Comparison of characteristics on the efficacy of the initial ^131^I treatment.

Characteristics	Successful ablation (*n* = 222)	Unsuccessful ablation (*n* = 187)	*χ* ^2^/*Z*-test	*p*-value
Gender			3.241	0.072
Men	47 (46.53%)	54 (53.47%)		
Women	175 (56.82%)	133 (43.18%)		
Age (years)			1.567	0.211
<45	129 (51.81%)	120 (48.19%)		
≥ 45	93 (58.13%)	67 (41.87%)		
Histopathological types			2.408	0.121
Papillary carcinoma	213 (53.52%)	185 (46.48%)		
Follicular carcinoma	9 (81.82%)	2 (18.18%)		
Primary focus			1.427	0.232
Unilateral	120 (57.14%)	90 (42.86%)		
Bilateral	102 (51.26%)	97 (48.74%)		
Tumor stage			3.182	0.204
I	175 (52.24%)	160 (47.76%)	1.589^a^	0.207^a^
II	28 (62.22%)	17 (37.78%)	1.891^b^	0.169^b^
III	19 (65.52%)	10 (34.48%)	0.083^c^	0.774^c^
Extrathyroidal extension			0.005	0.944
Without	110 (54.46%)	92 (45.54%)		
With	112 (54.11%)	95 (45.89%)		
LN metastasis			0.002	0.962
Without	36 (54.55%)	30 (45.45%)		
With	186 (54.23%)	157 (45.77%)		
Risk stratification of recurrence			6.184	0.045^*^
Low risk	16 (80.00%)	4 (20.00%)	4.679^d^	0.031*^,d^
Intermediate risk	107 (54.87%)	88 (45.13%)	6.120^e^	0.013*^,e^
High risk	99 (51.03%)	95 (48.97%)	0.576^f^	0.448^f^
Tumor size (cm)	1.20 (0.80–1.63)	1.30 (0.80–2.00)	−2.322	0.020^*^
RAIT dose (mCi)	120 (120–150)	150 (120–150)	−6.665	<0.001^*^
sTg (ng/ml)	0.70 (0.13–1.96)	8.69 (2.71–30.43)	−11.228	<0.001^*^
TSH (mU/L)	116.47 (89.68–161.37)	122.08 (95.86–167.63)	−0.773	0.439
TgAb (kU/L)	11.40 (10.00–81.60)	11.18 (10.00–43.33)	−0.197	0.844
sTg/TSH (mg/IU)	0.006 (0.001–0.018)	0.086 (0.018–0.254)	−10.651	<0.001^*^
sTg×TgAb	13.20 (5.09–33.80)	141.47 (49.00–506.99)	−13.121	<0.001^*^

^*^p-value <0.05. LN, lymph node; RAIT, radioactive iodine therapy; sTg, stimulated thyroglobulin; TSH, thyroid-stimulating hormone; TgAb, anti-thyroglobulin antibody. ^a,b^Tumor stage I group compared with stages Ⅱ and III groups, respectively. ^c^Tumor stage II group compared with stage III group. ^d,e^Low-risk group compared with intermediate- and high-risk groups, respectively. ^f^Intermediate-risk group compared with high-risk group.

### ROC Curves to Predict Unsuccessful Ablation

**Figure 2:**
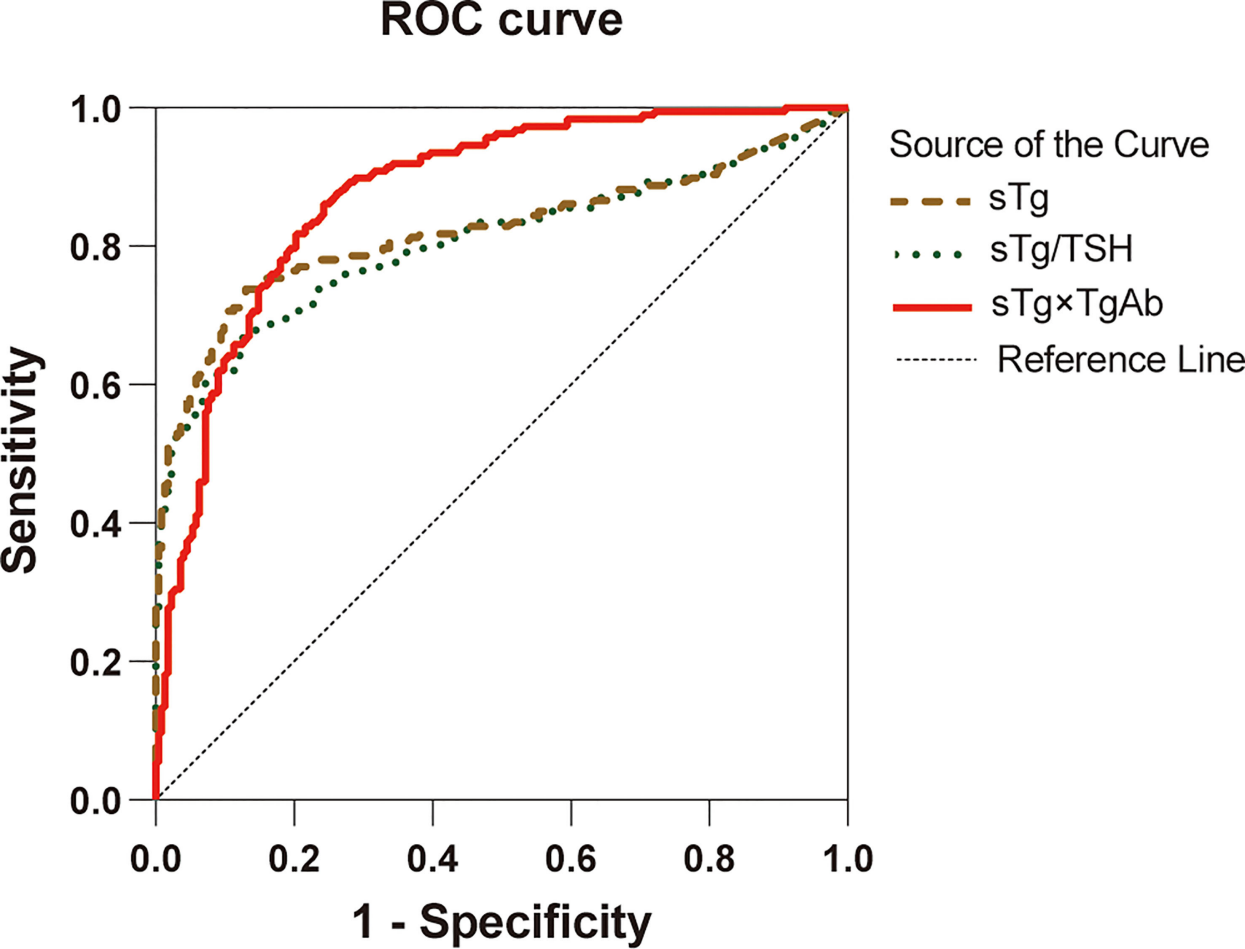
ROC curves for sTg, sTg/TSH ratio, and sTg×TgAb product before initial RAIT to predict unsuccessful ablation.

Preablation sTg, sTg/TSH ratio, and sTg×TgAb product were included in the ROC curve analysis (**Figure 2**), the area under ROC curve (AUC) to predict unsuccessful ablation was 0.822, 0.806, and 0.876, respectively, and the cutoff was 2.99 ng/ml, 0.029 mg/IU, and 34.18, respectively (**Table 2**). A pairwise comparison analysis of the ROC curves for the three predictors showed that the AUC for sTg×TgAb product was significantly greater than that for the other two factors (all *p* < 0.05). The AUC of the three factors was arranged in descending order as sTg×TgAb product, sTg, and sTg/TSH ratio, and the differences were statistically significant (all *p* < 0.05) (**Table 3**).

**Table 2:** Cutoff values* for predictors of successful ^131^I ablation with sensibility and specificity.

	Cutoff value^*^	Sensitivity (%)	Specificity (%)	AUC (95% CI)	*p*-value
sTg (ng/ml)	2.99	73.3	87.4	0.822 (0.777–0.867)	<0.001
sTg/TSH (mg/IU)	0.029	67.4	87.4	0.806 (0.760–0.851)	<0.001
sTg×TgAb	34.18	86.1	75.7	0.876 (0.843–0.910)	<0.001

^*^Value with maximum sensibility and specificity; AUC, area under the curve.

**Table 3:** Comparison among the ROC curves of preablation sTg, sTg/TSH ratio, and sTg×TgAb product.

Predictors	The difference of AUC (95% CI)	*Z*-test	*p*-value
sTg vs. sTg/TSH	0.016 (0.006–0.027)	3.016	0.003
sTg×TgAb vs. sTg	0.055 (0.016–0.094)	2.739	0.006
sTg×TgAb vs. sTg/TSH	0.071 (0.031–0.111)	3.448	<0.001

### Univariate and Multivariate Logistic Regression Analyses

Univariate logistic regression analysis found that the following clinical characteristics were not significantly related to the efficacy of ^131^I treatment: age, unilateral or bilateral primary foci, tumor stage, extrathyroidal extension, LN metastasis, and TSH level (all *p* > 0.05). On the other hand, gender as male, histopathological types as PTC, higher TgAb level, higher RAIT dose, bigger tumor size, intermediate-risk stratification of recurrence, sTg >2.99 ng/ml, sTg/TSH ratio >0.029 mg/IU, and sTg×TgAb product >34.18 were significantly related to unsuccessful ablation (all *p* < 0.1), and the above variables were included in the multivariate logistic regression analysis. The results showed that TgAb level, RAIT dose, sTg >2.99 ng/ml, and sTg×TgAb product >34.18 were independent risk factors for unsuccessful ablation (all *p* < 0.05). The odds ratio (OR) and 95% confidence interval (CI) of the above factors were 1.001 (1.000–1.002), 1.022 (1.007–1.037), 6.120 (2.202–17.008), and 4.200 (1.986–8.884), respectively (**Table 4**).

**Table 4:** Univariate and multivariate logistic regression analyses of tumor characteristics and therapeutic effect.

Characteristics	Univariate logistic analysis		Multivariate logistic analysis	
	OR (95% CI)	*p*-value	OR (95% CI)	*p*-value
Gender	1.512 (0.963–2.374)	0.073	1.526 (0.791–2.944)	0.207
Age (years)	0.774 (0.519–1.159)	0.211		
Histopathological types	0.256 (0.055–1.199)	0.084	0.351 (0.046–2.686)	0.313
Tumor size (cm)	1.257 (1.035–1.526)	0.021^*^	1.019 (0.768–1.352)	0.897
Primary focus	1.268 (0.859–1.872)	0.233		
Tumor stage		0.208		
II vs. I	0.664 (0.350–1.259)	0.210		
III vs. I	0.576 (0.260–1.275)	0.173		
Extrathyroidal extension	0.986 (0.668–1.455)	0.944		
LN metastasis	1.013 (0.597–1.719)	0.962		
Risk stratification of recurrence		0.003^*^		0.242
Intermediate vs. low	7.655 (1.001–58.556)	0.050^*^	1.859 (0.387–8.932)	0.439
High vs. low	3.613 (0.466–27.991)	0.219	1.076 (0.211–5.489)	0.930
TSH (mU/L)	1.001 (0.998–1.005)	0.362		
TgAb (kU/L)	1.001 (1.000–1.001)	0.051	1.001 (1.000–1.002)	0.006^*^
RAIT dose (mCi)	1.036 (1.025–1.047)	<0.001^*^	1.029 (1.011–1.048)	0.002^*^
sTg (ng/ml) (>2.99 vs. ≤2.99)	18.984 (11.380–31.669)	<0.001^*^	6.377 (2.269–17.924)	<0.001^*^
sTg/TSH (mg/IU) (>0.029 vs. ≤0.029)	13.747 (8.373–22.570)	<0.001^*^	1.219 (0.454–3.271)	0.694
sTg×TgAb (>34.18 vs. ≤34.18)	19.265 (11.506–32.255)	<0.001^*^	4.070 (1.928–8.588)	<0.001^*^

^*^p-value <0.05.

### Kaplan−Meier Analysis of PFS

**Figure 3:**
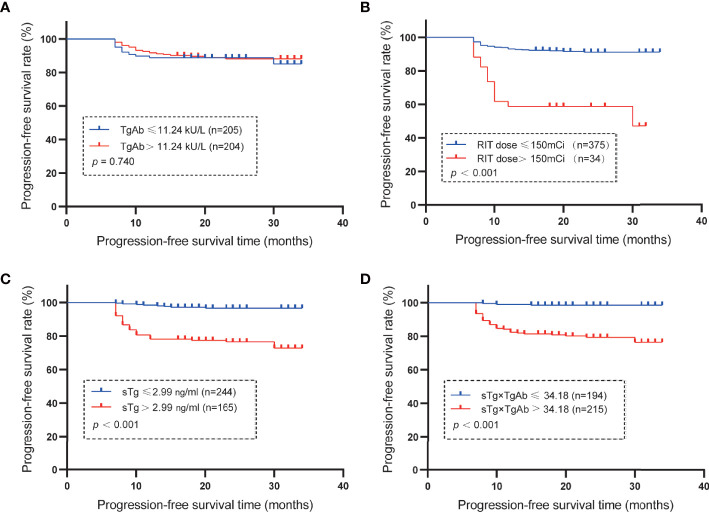
Mean PFS. **(A)** Mean PFS in the group of patients with TgAb ≤11.24 kU/L or >11.24 kU/L (the median of TgAb). The PFS was 30.96 months with TgAb ≤11.24 kU/L and 31.34 months for TgAb >11.24 kU/L (*p* = 0.740). **(B)** Mean PFS of the subset with RAIT dose ≤150 or > 150 mCi (the median of RAIT dose). The PFS was 31.91 months in patients with RAIT dose ≤150 mCi and 22.21 months with RAIT dose >150 mCi (*p* < 0.001). **(C)** Mean PFS in the subgroup of patients with sTg ≤2.99 or >2.99 ng/ml. The PFS was 33.26 months in patients with sTg ≤2.99 ng/ml and 28.06 months with sTg >2.99 ng/ml (*p* < 0.001). **(D)** Mean PFS in the group of patients with sTg×TgAb ≤34.18 or >34.18. The PFS was 33.64 months with sTg×TgAb ≤34.18 and 28.92 months for sTg×TgAb >34.18 (*p* < 0.001).

The median of TgAb levels and RAIT dose and the cutoff values of sTg and sTg×TgAb product were used as grouping standards to draw the Kaplan–Meier curves (**Figure 3**). Patients with TgAb ≤11.24 kU/L had a mean PFS of 30.96 months, while patients with TgAb >11.24 kU/L had a mean PFS of 31.34 months, without any significant difference (*p* = 0.740). The mean PFS of patients with RAIT dose ≤150 mCi was 31.91 months, which was significantly longer than that of patients with RAIT dose >150 mCi (mean 22.21 months, *p* < 0.001). The PFS of patients with sTg ≤2.99 ng/ml was significantly longer than that of patients with sTg >2.99 ng/ml (mean 33.26 vs. 28.06 months, *p* < 0.001), and that of patients with sTg×TgAb ≤34.18 was significantly longer than of those with sTg×TgAb >34.18 (mean 33.64 vs. 28.92 months, *p* < 0.001).

### Results of the TgAb-Negative and TgAb-Positive Groups

**Figure 4:**
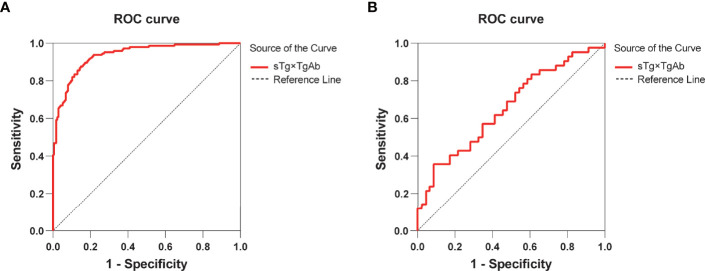
**(A)** ROC curve for sTg×TgAb product before initial RAIT to predict unsuccessful ablation of the TgAb-negative group. The cutoff for sTg×TgAb product was 36.80 (AUC: 0.937, 95% CI: 0.911–0.962) with 87.6% sensitivity and 85.2% specificity (*p* < 0.001). **(B)** ROC curves for sTg×TgAb product before initial RAIT to predict the unsuccessful ablation of the TgAb-positive group. The cutoff for sTg×TgAb product was 466.36 (AUC: 0.656, 95% CI: 0.542–0.770) with 35.7% sensitivity and 91.3% specificity (*p* = 0.012).

**Figure 5:**
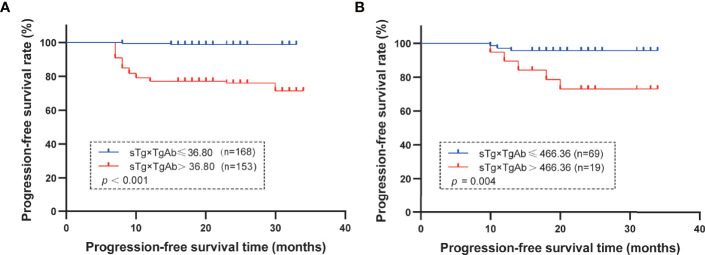
**(A)** TgAb-negative group: mean PFS in the group of patients with sTg×TgAb ≤36.80 or >36.80. The PFS was 32.74 months with sTg×TgAb ≤36.80 and 27.82 months for sTg×TgAb > 36.80 (*p* < 0.001). **(B)** TgAb-positive group: mean PFS by group of patients with sTg×TgAb ≤36.80 or >36.80. The PFS was 33.01 months with sTg×TgAb ≤466.36 and 28.84 months for sTg×TgAb >466.36 (*p* = 0.004).

According to the results of the multivariate logistic regression analysis of the 409 patients, TgAb level, RAIT dose, sTg, and sTg×TgAb product were independent risk factors for unsuccessful ablation. These factors were included in the clinical evaluation of the TgAb-negative and TgAb-positive groups (**Table 5**). We found that only sTg×TgAb product was significantly lower in patients with successful ablation compared with those with unsuccessful ablation in both groups (*p* < 0.05). The parameter was included in the ROC curve analysis to predict the unsuccessful ablation of both groups (**Figure 4**). The cutoffs were 36.80 (AUC: 0.937) and 466.36 (AUC: 0.656), respectively, the sensitivities were 87.6% and 35.7%, and the specificities were 85.2% and 91.3%, respectively. According to the Kaplan–Meier curves of the two groups, the PFS of patients with sTg×TgAb ≤36.80 was significantly longer than that of patients with sTg×TgAb >36.80 (mean 32.74 vs. 27.82 months, *p* < 0.001) in the TgAb-negative group. In the TgAb-positive group, the PFS of patients with sTg×TgAb ≤466.36 was significantly longer than that of patients with sTg×TgAb >466.36 (mean 33.01 vs. 28.84 months, *p* = 0.004) (**Figure 5**).

**Table 5:** Comparison of the three independent risk factors for unsuccessful ablation of the TgAb-negative group (≤115 kU/L) and the TgAb-positive group (>115 kU/L) on the efficacy of the initial ^131^I treatment.

Characteristics	TgAb ≤115 kU/L				TgAb >115 kU/L			
	Successful ablation (*n* = 176)	Unsuccessful ablation (*n* = 126)	*Z*-test	*p*-value	Successful ablation (*n* = 46)	Unsuccessful ablation (*n* = 42)	*Z*-test	*p*-value
TgAb (kU/L)	10.00 (10.00–112.60)	10.00 (10.00–85.64)	−1.297	0.195	241.25 (129.50–2,647.00)	407.50 (123.40–3,611.00)	−3.312	0.001^*^
RAIT dose (mCi)	120 (100–200)	150 (120–200)	−6.889	<0.001^*^	120 (100–150)	120 (120–200)	−1.547	0.122
sTg (ng/ml)	0.80 (0.04–21.77)	12.84 (0.04–500.20)	−13.329	<0.001^*^	0.10 (0.04–8.19)	0.21 (0.04–206.00)	−0.908	0.364
sTg×TgAb	9.60 (0.40–217.70)	147.32 (1.60–7,200.54)	−13.473	<0.001^*^	38.10 (8.03–3,547.19)	86.28 (6.59–55,306.17)	−2.515	0.012^*^

^*^p-value <0.05.

## Discussion

Under the concept of comprehensive multidisciplinary treatment, selective ^131^I therapy has become one of the main methods of postoperative adjuvant treatment to improve the prognosis of DTC patients (6, 21). However, the success rate of the initial ^131^I treatment after DTC surgery is varied (8, 22–25). Therefore, analyzing the factors that affect the efficacy of RAIT and predicting the efficacy and prognosis of RAIT in advance can be valuable in stratifying the risk of patients after DTC surgery and adjusting the treatment plan.

To avoid the interference of TgAb to sTg, the previous studies on the factors affecting the efficacy of RAIT selected TgAb-negative patients as the research subjects (4, 7–12). However, even if TgAb is negative, it affects sTg measurements (18). The present study included TgAb-negative and TgAb-positive patients and studied the influencing factors and the predictors of the efficacy and prognosis of RAIT in the total sample and the two groups. According to the correlation analysis between sTg and TgAb, the present study corrected their mutual influence by sTg×TgAb product and tested its ability to predict the efficacy and prognosis of RAIT.

The clinical characterization demonstrated that preablation sTg and sTg/TSH ratio are significantly related to the efficacy of RAIT, which is consistent with the results of previous studies (13–15, 17). In addition, statistical analysis revealed that sTg×TgAb product, tumor size, risk stratification for recurrence, and RAIT dose are significantly related to the efficacy of RAIT, while the remaining clinical characteristics were similar between the successful and unsuccessful ablation groups. It has been reported that ^131^I dose is an important factor affecting the therapeutic effect of RAIT ablation, but the results of the studies were different. Kim et al. (26) stated that high doses of iodine treatment are associated with a high success rate, while Trevizam et al. (12) got a result similar to this study that the RAIT dose of the unsuccessful ablation group was significantly higher than that of the successful ablation group. The results of the present study also indicated that higher RAIT dose was an independent risk factor for unsuccessful ablation. The risk of unsuccessful ablation increases by 1.029-fold for every increase of 1 mCi in the RAIT dose. These differences may be attributable to the difference in grouping methods or the research objects.

To confirm the predictive value of sTg×TgAb product, preablation sTg, sTg/TSH ratio, and sTg×TgAb product were included in the ROC curve analysis, and the results showed that these parameters predicted the therapeutic effects of ^131^I treatment. When preablation sTg was ≤2.99 ng/ml, 79.51% of DTC patients receiving RAIT are probable to succeed in ablation. About 75.98% of patients are likely to succeed in ablation when sTg/TSH ratio was ≤0.029 mg/IU. Comparing our results with those of Wang et al. (8) and Trevizam et al. (12), we found that the cutoff values, sensitivities, and the AUC of sTg and sTg/TSH ratio obtained in this study are the lowest, and the specificities are the highest. These inconsistent results may be related to the different inclusion criteria or grouping methods across studies. Moreover, the results of this study showed that when sTg×TgAb product was ≤34.18, up to 86.60% of DTC patients receiving RAIT is probable to succeed in ablation, which was higher than that of the sTg and sTg/TSH ratio. Subsequently, we compared the ROC curves for these three predictors and found that the AUC for sTg×TgAb product was the highest and significantly greater than that for the sTg and sTg/TSH ratio, indicating that the predictive ability of the sTg×TgAb product is better than the sTg and sTg/TSH ratio. Compared with the sTg and sTg/TSH ratio, the sTg×TgAb product has a higher sensitivity and lower specificity. In clinical application, we can reduce the false-positive rate of the sTg×TgAb product by taking all of these predictors into consideration.

In recent years, TgAb has been gaining increasing attention as a critical indicator of the surveillance of DTC. The ATA guidelines indicated that the increased TgAb levels after total thyroidectomy and RAIT suggested an increased risk of recurrence (6). The results of univariate and multivariate logistic regression analyses showed that the risk of unsuccessful ablation increases by 1.001-fold for every 1 kU/L increase in TgAb level. Combining with the result, the influence of TgAb on the prognosis of DTC was further confirmed. In addition, the results indicated that the risk of unsuccessful ablation in patients with sTg >2.99 ng/ml was 6.377-fold than that of patients with sTg ≤2.99 ng/ml, and the risk of unsuccessful ablation in patients with sTg×TgAb >34.18 is 4.070-fold than that of sTg×TgAb ≤34.18.

After a median follow-up of 23 months, we found that the PFS of patients with sTg >2.99 ng/ml was significantly shorter than that of patients with sTg ≤2.99 ng/ml similar to patients with sTg×TgAb >34.18 compared with patients with sTg×TgAb ≤34.18. This phenomenon further confirmed the predictive value of preablation sTg level and sTg×TgAb product for the prognosis of ^131^I treatment. According to the ATA guidelines, TgAb is an alternative tumor marker in TgAb-positive DTC patients (6). However, in this study, no significant difference was observed between the mean PFS of patients with TgAb ≤11.24 kU/L and that of patients with TgAb >11.24 kU/L, indicating TgAb cannot be an independent predictor of the prognosis of RAIT in the total population.

The analysis of the TgAb-negative and TgAb-positive groups showed that sTg×TgAb product predicts the efficacy and prognosis of RAIT and whether patients are in TgAb-negative or TgAb-positive status. Nevertheless, sTg was significantly associated with the efficacy of RAIT only in the TgAb-negative group, and TgAb showed a significant association with the efficacy of RAIT only in the TgAb-positive group, which further confirmed that sTg×TgAb product is a universal predictor.

The limitation of this study is the small sample size, especially of the TgAb-positive group. The selection bias cannot be avoided due to the limitation of retrospective analysis and the short follow-up duration. Because the pre-ablation TSH level is difficult to control, we did not select patients with the same TSH level even though it could affect sTg level (2, 27, 28). However, sTg/TSH ratio was included in this study, and its predictive ability was compared with the other predictors.

In summary, this study confirmed that before the first ^131^I treatment following total thyroidectomy, sTg×TgAb product could predict the efficacy and prognosis of ^131^I therapy for both TgAb-negative and TgAb-positive DTC patients. It can be used as a clinical reference indicator, and for patients with sTg×TgAb above the cutoff value, the risk can be restratified and the treatment plan improved. In future studies, we will expand the sample size and conduct prolonged surveillance to verify the reliability of this indicator further.

## Data Availability Statement

The original contributions presented in the study are included in the article/supplementary material. Further inquiries can be directed to the corresponding authors.

## Author Contributions

MP and XL contributed to the concept and design of this study. MP collected data, completed statistical analyses, and drafted the manuscript. MJ and ZL critically revised the manuscript. All authors listed have made a substantial, direct, and intellectual contribution to the study and approved it for publication.

## Conflict of Interest

The authors declare that the research was conducted in the absence of any commercial or financial relationships that could be construed as a potential conflict of interest.

## Publisher’s Note

All claims expressed in this article are solely those of the authors and do not necessarily represent those of their affiliated organizations, or those of the publisher, the editors and the reviewers. Any product that may be evaluated in this article, or claim that may be made by its manufacturer, is not guaranteed or endorsed by the publisher.
